# Ultrasonic Vocalizations of Male Mice Differ among Species and Females Show Assortative Preferences for Male Calls

**DOI:** 10.1371/journal.pone.0134123

**Published:** 2015-08-26

**Authors:** Kerstin Musolf, Stefanie Meindl, Angela L. Larsen, Matina C. Kalcounis-Rueppell, Dustin J. Penn

**Affiliations:** 1 Department of Integrative Biology and Evolution, Konrad Lorenz Institute of Ethology, University of Veterinary Medicine, Vienna, Austria; 2 Department of Biology, Brooklyn College, Brooklyn, New York, United States of America; 3 Department of Biology, University of North Carolina at Greensboro, Greensboro, North Carolina, United States of America; University of Arkansas, UNITED STATES

## Abstract

Male house mice (*Mus musculus*) emit ultrasonic vocalizations (USVs) during courtship, which attract females, and we aimed to test whether females use these vocalizations for species or subspecies recognition of potential mates. We recorded courtship USVs of males from different *Mus* species, *Mus musculus* subspecies, and populations (F1 offspring of wild-caught *Mus musculus musculus*, *Mus musculus domesticus* (and F1 hybrid crosses), and *Mus spicilegus*), and we conducted playback experiments to measure female preferences for male USVs. Male vocalizations contained at least seven distinct syllable types, whose frequency of occurrence varied among species, subspecies, and populations. Detailed analyses of multiple common syllable types indicated that *Mus musculus* and *Mus spicilegus* could be discriminated based on spectral and temporal characteristics of their vocalizations, and populations of *Mus musculus* were also distinctive regardless of the classification model used. Females were able to discriminate USVs from different species, and showed assortative preferences for conspecific males. We found no evidence that females discriminate USVs of males from a different subspecies or separate populations of the same species, even though our spectral analyses identified acoustic features that differ between species, subspecies, and populations of the same species. Our results provide the first comparison of USVs between *Mus* species or between *Mus musculus* subspecies, and the first evidence that male USVs potentially facilitate species recognition.

## Introduction

Ultrasonic vocalizations (USVs) in rodents have been recognized for more than 100 years (in theory: [1], first recording [2]), and recent analyses of the spectrographic features of the USVs of male laboratory house mice (*Mus musculus*) have revealed surprising complexity [3]. Male house mice emit USVs during courtship, and their vocalizations may facilitate mating [4]. Male USVs are attractive to females (wild [5] and laboratory mice [6]), and it has been suggested that females use USVs to obtain information about male quality and compatibility [7]. For example, females may use male USVs for individual recognition, kin recognition, and inbreeding avoidance. In wild-derived house mice, several features of male USVs are more similar between brothers than among non-siblings [8], and females show preferences for playbacks of USVs from unrelated males compared to their siblings [5]. Similarly, females may also use male USVs to avoid interspecific or subspecific hybridization [7, 9]. Courtship vocalizations in many species have been found to contain species-specific calls and are used to avoid hybridization [10]. Recent evidence suggests that vocalizations facilitate species recognition in Neotropical singing mice (*Scotinomys spp*. [11, 12]). Therefore, we aimed to test whether female house mice (*M*. *m*. *musculus)* recognize and show assortative preferences for the USVs of males from their own species compared to other mice—including a closely related species, subspecies, and hybrids, and whether spectral and temporal features of the USVs of these groups differ.

Previous studies on house mice suggest that male courtship USVs function to coordinate mating behavior (reviewed in [7]). USVs appear to signal male sexual arousal [13], help to keep females in close proximity during courtship [4], and facilitate copulation behavior [14]. Females potentially use male USVs to recognize male social status, as dominant males call at higher rates than subordinates [15, 16] and social defeat leads to reduced calling [17]. The USVs of wild male house mice also contain signatures of individuality [8] and females prefer the calls of unrelated males versus siblings (kin recognition) [5]. Male USVs contain strain-specific features, even when cross-fostered [18–20], and females learn the USVs of their parents and prefer songs that differ from their parents’ (classical negative imprinting) [21]. Thus, female mice may use male USVs, in addition to chemical signals (e.g., [22–24]), to select their mates. However, there has been no test of whether female house mice use male USVs for species or subspecies recognition or whether they show assortative preferences (differential attraction to calls of conspecific males) [7, 9]. Furthermore, USV studies have primarily been conducted on inbred laboratory strains and there have been no comparisons of USVs among *Mus* species. More studies on wild rodents are needed because their vocalizations show more complexity than their laboratory counterparts (*Peromyscus californicus* [25], *M*. *m*. *musculus* [26]).

There are more than 23 species in the *Mus* genus and the phylogeny of the group is well resolved [27, 28]. *M*. *musculus*, the species from which laboratory mice were derived, is closely related to *M*. *spicilegus* (the mound-building mouse)–both are members of the *M*. *musculus* species group—and while they share striking morphological similarities, they are genetically and behaviorally distinct [29–31]. *M*. *musculus* and *M*. *spicilegus* often live sympatrically, and though hybrids have been produced in the laboratory, behavioral preferences for and physiological responsiveness to conspecifics indicate strong precopulatory isolation mechanisms between these species [32]. In contrast, the taxonomic status of the *Mus musculus* subspecies cluster is complex and not well resolved [30, 33, 34]. Phylogenetic analyses of mitochondrial DNA indicate that there are five subspecies in this group [35], including *M*. *m*. *musculus* and *M*. *m*. *domesticus* [30, 33, 34]. These two subspecies hybridize in their secondary contact zone [36], but they remain genetically distinct. Hybrid males between these two subspecies often suffer from infertility due to genetic incompatibilities [37–39], which is why these subspecies were previously classified as separate species, *M*. *musculus* and *M*. *domesticus*. In allopatry, behavioral isolation of subspecies is incomplete because, while *M*. *m*. *musculus* shows assortative odor and partner preferences, *M*. *m*. *domesticus* is indiscriminate [34, 40]. However, mice living near the hybrid zone display accentuated urinary signals and stronger assortative odor preferences for their own subspecies (reproductive character displacement) [41, 42], which suggests reinforcement selection through assortative mate choice.

To test whether USVs in house mice mediate species, subspecies, and population recognition, we recorded the courtship USVs of males from eight different wild-derived house mouse populations, all members of the *M*. *musculus* species group (F1 offspring from wild-caught *M*. *m*. *musculus* from 4 locations, *M*. *m*. *domesticus*, as well as two colony-reared hybrid lines of *M*. *m*. *musculus* x *M*. *m*. *domesticus* crosses, and *M*. *spicilegus*). We analyzed spectral and temporal parameters of male USVs to determine whether *Mus* species, *M*. *musculus* subspecies and *M*. *m*. *musculus* populations can be discriminated using classification models. In addition, we conducted playback experiments to assess whether females are able to discriminate between courtship calls produced by males from different species, subspecies or other populations from the same species. If male USVs facilitate species recognition and females show assortative preferences for male USVs, then these vocalizations potentially facilitate hybridization avoidance and speciation [43].

## Methods

### Subjects and housing

Animals in our study were colony-bred offspring of wild-caught house mice and mound-building mice. House mice were originally trapped at four sites surrounding Vienna, Austria, including *Safaripark* (48°18’22”N, 16°43’48”E), *Schottenhof* (48°14’54”N, 16°15’32”E), *VetMedUni* (48°15’22”N, 16°25’55”E), and *KLIVV* (48°12’38”N, 16°16’54”E), with different degrees of genetic variability in the founders [44]. Distinct populations (F1–F3) were subsequently maintained via outbreeding and abbreviated as *M*.*m*.*m*.1, *M*.*m*.*m*.2, *M*.*m*.*m*.3, and *M*.*m*.*m*.4. The *M*. *m*. *domesticus* population was represented by F3 offspring of wild-caught mice from the Massif Central, France (henceforth *M*.*m*.*d*.) [45]. Hybrids were F1 crosses between *M*.*m*.*d*. females x *M*.*m*.*m*. males from our colony (henceforth Hybrid 1: *M*.*m*.*d*. x *M*.*m*.*m*.1, Hybrid 2: *M*.*m*.*d*. x *M*.*m*.*m*.3). Herein, *M*. *musculus* (*M*.*m*.) will refer to all populations of *M*. *musculus* (*M*.*m*.*m*.1, *M*.*m*.*m*.2, *M*.*m*.*m*.3, *M*.*m*.*m*.4, *M*.*m*.*d*, Hybrid 1, and Hybrid 2). *M*. *spicilegus* individuals were F1 offspring of wild mound-building mice caught at two different locations in Western Slovakia (Bohelov: 47°54’26” N, 17°41’58” E; Sasa 48°03' N; 17°25'E) (henceforth M.s.).

House mice were reared in mixed-sex family groups until weaning at 21 days of age. After weaning, males were individually housed to prevent fighting, and females were kept as sister pairs in type II cages (size: 26.5 x 20.5 x 18 cm, plus high stainless steel covers, mesh width 1 cm) with bedding and nesting material (ABEDD, Vienna, Austria: aspen wood chips and shavings) and shelter (paper rolls). Mound-building mice were kept in same-sex as well as in mixed-sex litter groups in interconnected type II cages (size: 26.5 x 20.5 x 18 cm, plus high stainless steel covers, mesh width 1 cm) with bedding and nesting material (ABEDD, Vienna, Austria). Home cages were kept at standard conditions (mean temperature 20 ± 1°C and 12:12 h light: dark cycle; lights on at 07:00 a.m.). Food (Altromin, Lage, Germany) and water were provided *ad libitum*. All experimental mice were sexually mature (> 8 weeks). Animals were not acoustically isolated prior to the experiment (except for *M*.*s*., which were bred in a different colony room) and were kept within the same colony room.

### Social experience

We ensured that all *M*.*m*. experimental males were socially or sexually experienced prior to experimental trials: before recording, all males had previously participated in a mate choice experiment, which involved direct interaction with a female over four weeks (*M*.*m*.*m*.), or received social experience by being systematically exposed to both male and females through a fence when sexually mature (*M*.*m*.*d*., Hybrid 1 & 2; method described in detail in [5]). As males of *M*.*s*. continued to live in mixed sex family groups through adulthood, no further social experience treatment was performed.

### Urinary stimulus collection

To trigger male USV emission for recording, fresh female urine was used as a stimulus [46]. Female urine was collected from donor females placed on a surface covered with clean aluminum foil. Handling was usually sufficient to induce urination, but urination could also be triggered by ventral stroking in an anterior-to-posterior direction [47]. Urine was collected immediately and fresh urine was subsequently pipetted onto a clean cotton swab (storage ≤ 5 min, volume ≥ 60 μl). In addition, soiled bedding (12 g) of the same female was collected from her home cage and presented simultaneously by placing it in front of the test animal. Urine and bedding were derived from different unfamiliar, randomly selected adult females from our captive mouse colony regardless of their reproductive stage, as there is mixed evidence that female estrus affects male USV emission [47, 48].

To enhance female responsiveness to male USVs during acoustic playback experiments, we collected urine from several males, which we pooled and presented to females, as an additional stimulus. Equal amounts of fresh urine (5 μl per male) were mixed in a 0.2 ml PCR tube and stored at -20°C. Here, due to the number of males (10), it was not possible to simultaneously collect fresh aliquots of all males for each pool. A urinary pool consisted of aliquots of 10 unfamiliar male mice of each population or species (50 μl each). For tests including *M*.*s*., the urine pool for the comparing stimuli (*M*.*s*. and *M*.*m*.*m*.1) was reduced to 12.5 μl (1.25 μl per male), as the amount of urine obtained from *M*.*s*. was generally low. Experimenters wore clean gloves at all times and metal foil and gloves were exchanged immediately after each urine collection.

### Apparatus, calibration and recording procedure

Recordings were made in an acoustically isolated recording room with no other animals present. A sound-insulated chamber (65 cm x 65 cm, inside lining: acoustic pyramid foam (5 cm)) was used for recording USVs from individual males within their home cages. The insulated lid provided an opening for the condenser microphone (UltraSoundGate CM16/CMPA, 15–180 kHz, flat frequency response (± 6 dB) between 25 and 140 kHz; Avisoft Bioacoustics, Berlin, Germany) which was fixed 20 cm above the cage in the middle of the box. For monitoring USVs, we used an UltraSoundGate 116 (Avisoft Bioacoustics) and an external soundcard (Edirol UA-101, 24-Bit/192 kHz 10-in/10-out Hi—SPEED USB (USB 2.0) audio interface for multitrack computer recording). Settings included sampling rate at 250 kHz and a format of 16 bit. Recordings were transferred to a sound analysis system (Avisoft-SASLab Pro, Version 4.40, Avisoft Bioacoustics) for processing. Spectrograms were generated with a fast Fourier transformation (FFT)-length of 512 points and a time window overlap of 50% (100% Frame, FlatTop window). A noise reduction by 90 dB below– 52 dB was used to reduce background noise whilst not compromising automated call detection. We calibrated recording equipment by recording a pure tone of 440 Hz (commercially available tuning fork) and comparing the frequency in the spectrogram with the actual frequency recorded.

Before each recording session, a clean cage lid was put on each male’s home cage (type II; without food pellets and water bottle to reduce sound interference). The cage with the mouse was placed in the center of the recording chamber. Following a 5 min habituation period, test trials began after the introduction of female urine and soiled-bedding stimuli. Recording trials lasted 30 min each after stimuli introduction and after each trial, the recording chamber was cleaned using a handheld vacuum cleaner. The temperature of the testing room was held constant at 20.4° ± 0.9°C. During recording, no one was present in the recording room.

### Experiment 1—USV variability among populations and species of male mice

**Fig 1:**
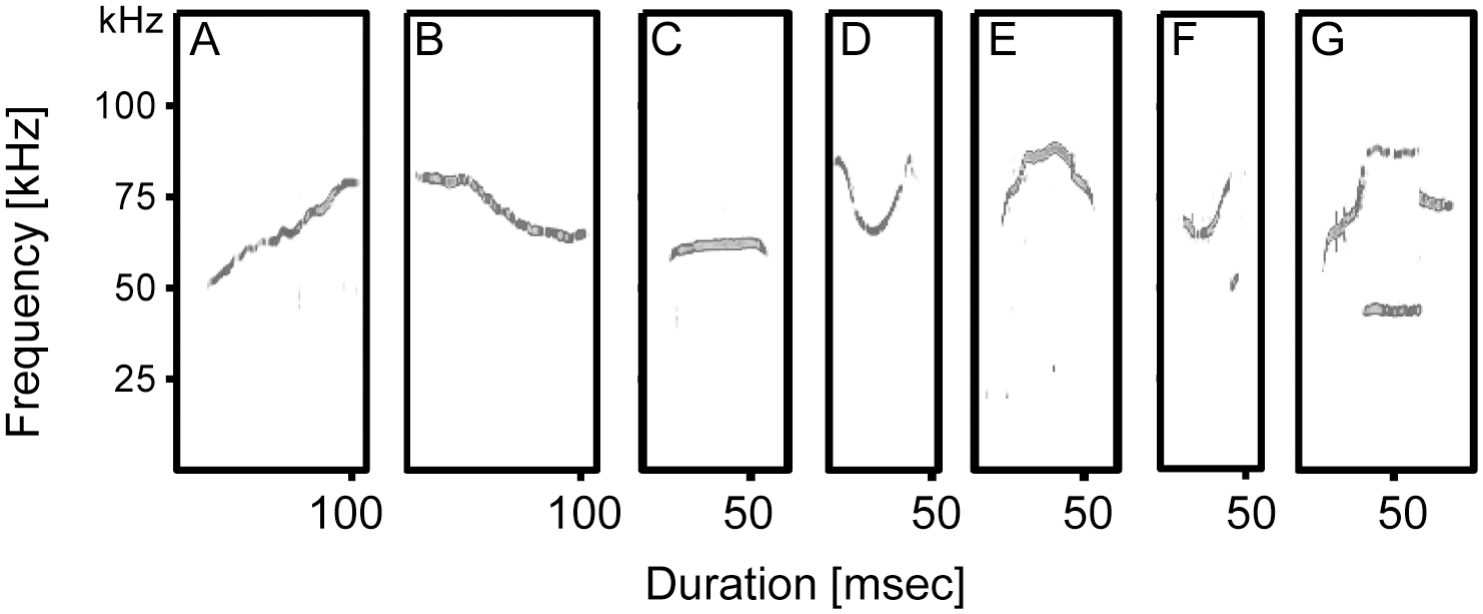
Spectrograms of the seven ultrasonic syllable types. Syllable types are classified according to spectrographic parameters, e.g., start, end, and center frequency, frequency at peak energy and duration (details in Methods). (A) Frequency Upsweep (B) Frequency Downsweep (C) Constant Modulated (D) U-Shaped (E) U-Shaped Inverted (F) 1-Frequency-Step (G) 2-Frequency-Step

We compared USV characteristics of males from different mouse populations, subspecies and species. In addition to the presence or absence of vocalizers within populations, distinct USV parameters and syllables (discrete sound units separated by silence from each other [3, 25]; Fig 1), spectrographic features, and vocal repertoire size were compared. Altogether 127 socially experienced, mature males (255.4 ± 97.3 SD d of age) were recorded until a sample size of 10 males per population was reached. Due to high proportions of non-vocalizing males and limited number of experimental animals, samples sizes were lower in the hybrid lines (Hybrid 1: N = 5; Hybrid 2: N = 7) and in the *M*.*m*.*d*. population (N = 4). Only vocalizers were used for further spectrographic analyses (N = 66; 269.3 ± 103.8 SD d of age).

Despite prior noise reduction (by 90 dB below– 52 dB), recordings still contained a considerable amount of ‘non-USV’ sound, and this background noise was manually removed from spectrograms. Parameters were determined automatically (Avisoft-SASLab Pro; version 4.40), including minimum and maximum frequency, peak frequency, peak amplitude, entropy and bandwidth, which were derived from start, center, and end spectrum of the entire syllable. Peak frequency is defined as the frequency at the location of maximum amplitude. Peak amplitude is defined as the point with the highest intensity within the spectrum. Entropy is a measure of the bandwidth and uniformity of the spectrum of a sound; tonal (whistle-like sounds) usually have low entropy (< 0.3), while broad-band (noisy) sounds have higher entropies (> 0.4). Bandwidth is calculated as the frequency difference between maximum and minimum frequency. Mean call duration was measured as a temporal parameter of syllables.

The first 100 syllables produced by each male were analyzed in detail, and based on characteristic spectrographic shape and parameters, syllables were classified into 7 categories as follows: (1) **Frequency Upsweep**: continuous increase in peak frequency ≥ 10kHz frequency modulation; (2) **Frequency Downsweep**: continuous decrease in peak frequency ≥ 5kHz frequency modulation; (3) **Constant Modulated**: ≤ 2kHz frequency modulation; (4) **U-Shaped**: U-shape wave ≥ 4kHz frequency modulation; (5) **U-Shaped Inverted** (Chevron): inverted-U shape ≥ 4kHz frequency modulation; (6) **1-Frequency-Step**: instantaneous frequency step, like a vertical discontinuity with no time gap; (7) **2-Frequency-Step**: 2 instantaneous frequency steps [49–51] (Fig 1A–1G). The total number of distinct syllable types was used to estimate a male’s repertoire size.

To examine between-species and between-population differences in extracted spectral and temporal characteristics of USVs, we calculated individual mean values for all USV types of a given species or population. To reduce the dimensionality of the 25-variable data set, we extracted 5 principal component axes (2 frequency, 1 amplitude, 1 bandwidth, and 1 entropy) from spectral and temporal characteristics. Prior to the PCA analysis we identified multivariate outliers in the dataset, using squared standardized distance. This measure allowed us to focus on potentially influential points to consider for removal. We removed the 12 most extreme outliers. To standardize across different measurement scales, a correlation matrix was used in the PCA.

To assess differences in USV characteristics among groups, we made the following comparisons. First, we compared *M*.*m*. with *M*.*s*. Second, we compared subspecies of *M*.*m*. (all populations of *M*.*m*.*m*. and the population of *M*.*m*.*d*.). The subspecies comparison was conducted without hybrids. Third, we compared all four populations within the *M*.*m*.*m*. subspecies. Lastly, we compared all populations, at all taxonomic levels, with each other, for a total of 8 groups. We performed all comparisons considering all USV types combined and considering each call type independently.

#### Data analyses and statistical tests

We confirmed the assumptions of models and tests before applying, and when data were non-normal and could not be transformed, nonparametric tests were applied. To test whether populations differed with respect to their ratio of vocalizing/non-vocalizing males, χ^2^ tests with false discovery rate (FDR) controls were conducted. A binominal test was applied, setting the expected proportion to 0.5 (50%) to analyze the frequency of vocalizers/non-vocalizers within populations. Latency until first ultrasonic call was measured as a temporal feature of vocalizations. A nonparametric Kruskal-Wallis-H test was applied followed by Mann-Whitney U tests to compare latencies of the different populations. The total number of uttered syllables per individual per 30 minutes recording period was counted by visual inspection of spectrograms. An effect of age on latency and / or USV emission rate was tested via Spearman rank correlation for nonparametric data. Repertoires were compared using nonparametric Kruskal-Wallis-H, followed by Mann-Whitney U tests. In order to test the classification of populations based on USV features, we compared two classification methods: support vector machine (SVM) and discriminant function analysis (DFA). We used the 5 principal component scores and the mean duration variable as a reduced dataset in separate analyses. For the species comparison, we also used the raw, 25 variable data set. For the SVM, each population dataset was randomly divided into training (80%) and testing (20%) datasets. SVM models were separately tuned with the respective training dataset to obtain the best gamma and cost parameters for each model using 10-fold cross validation. Data from all USV types were included in the analysis. DFA and multivariate analysis of variance (MANOVA) was used to test for differences in the USV characteristics among populations with populations used as a classification factor. For the DFA, Wilk’s lambda was used to estimate discrimination among individuals and an F-test was used to determine its significance [52]. DFA cross-validation classification rates are presented for comparison with SVM classification rate. For all groups where SVM and DFA classification occurred, we also compared the variables using either Kruskal-Wallis-H or applied Mann-Whitney U tests. All statistical analyses were performed using SPSS (version 15.01 for Windows) and R (3.0.2) [53]. Results are reported as mean ± SE, unless otherwise specified. In all cases p < 0.05 was considered to be statistically significant, and tests were two-tailed. FDR was used to control for Type I errors due to multiple testing [54].

### Experiment 2 –Female discrimination of male USV playbacks

We tested females for their ability to discriminate USVs of different mouse populations, subspecies and species using USV playbacks. The females in our choice experiments originated from a single *M*. *musculus* population (*M*.*m*.*m*.1), and mice from this population were used in previous USVs experiments [5]. Females were tested in or near estrus, which was determined by vaginal smears [55, 56] collected 5–7 h before the trial to prevent handling stress which might affect the experiment. To synchronize and increase the number of estrous females, 12 g of male soiled bedding (unrelated to the females and the subsequent testing stimuli) was introduced as a priming stimulus to female home cages 3 days prior to their use in the experiment [57]. The choice apparatus was constructed from a type III cage (42.5 x 27 x 20 cm), specially modified for assessing females’ attraction to playbacks through one of two speakers at one end (‘Speaker zone’) (see S1 Fig). Individual females were placed at the opposite end (‘Neutral zone’) from which they could move into one of two equal-sized compartments, which both contained a fenced speaker for playing ultrasound recordings (Ultrasonic Dynamic Speaker Magnat, dominant frequency range 1–55 kHz; impedance 4 ohm, Avisoft Bioacoustics, Berlin, Germany; using an external soundcard (Edirol UA-101) covering ultrasonic frequency ranges) and were separated by acoustic foam (Pur Skin, 10 mm, SONATECH). Acoustic insulation (pyramid foam, 5 cm) ensured that playbacks played on one side were only heard in the corresponding arm and ‘Neutral zone’, allowing females to hear playbacks from both compartments at the onset of the experiment. Signal quality was verified by spectral comparison of recordings from original and played-back USVs. For analyzing females’ relative attraction, compartments were further divided into different proximity zones: ‘Fence’ (mice in contact with the fence in front of the speakers); ‘Speaker zone’ (area 0–9 cm from the speaker/fence); ‘Middle zone’ (area 9–18 cm apart); and ‘Neutral zone’. As previously mentioned, females show limited interest when playbacks were the only stimulus and therefore, male urine was also provided as an ‘enhancing stimulus’ [58]. This stimulus consisted of two different pools of urine of 10 males (unfamiliar and unrelated to females), which were pipetted on filter paper (Whatman 3MM Chr, 0.34 mm thick, 4 x 4 cm) in front of the speakers. The urine stimuli in each compartment were identical in volume and composition, i.e., urine from males belonging to the species (or population) of *both* test populations was placed on both sides to prevent biasing the results. After females were placed in the ‘Neutral zone’, habituation lasted as long as the animal explored both compartments and returned to the ‘Neutral zone’ (all females did) after which playback was initiated.

Playbacks consisted of 310 sec of previously recorded uttered syllable bouts, and were standardized by the number of syllables (399 ± 3 SD, Avisoft SASLab Pro; version 4.40) to avoid preferences on the basis of performance-related traits [59]. Artificial inter-syllable durations were the same as those found in mice (< 1 sec, range 0.03–0.94), and duration between each individual phrase (sequence of syllables uttered in close succession) was 1 sec. Playback amplitude was standardized for both sides.

Experiments were digitally videotaped (Sony Handycam DCR-SR30) and analyzed blindly regarding female identity and trial number (using ‘The Observer 7.0’, *Noldus*). Retention times in the designated areas of the apparatus (Neutral zone, Middle zone, Speaker zone and Fence) were measured and compared. Self-grooming and sniffing at the enhancing urinary stimulus and their relative proportions were measured as behavioral parameters, providing further indicators for mating preferences [60].

As a positive control, we confirmed that females are generally attracted to male USVs by testing a USV playback versus silence (no-USV playback). Females (*M*.*m*.*m*.1, N = 10; 209 ± 72 SD d of age at trial) spent significantly more time at the fence in front of the USV playback speaker and significantly more time in the USV playback zones compared to the side with no USV (Wilcoxon signed-rank test, Median [sec] (interquartile range IQR): Fence: 36.8 (27.7–53.8) vs. 27.7 (5.3–36.9), Z = -1.376, p = 0.169; Fence + Speaker zone: 79.9 (48.5–171.7) vs. 48.6 (8.7–80.9), Z = -1.998, p = 0.047; Fence + Speaker + Middle zone: 102.4 (77.1–179.5) vs. 63.9 (14.8–105.4), Z = -2.293, p = 0.022) (S2A Fig).

Population playbacks (pooled USVs) were composed of a collection of alternating calls of different males to reflect the complete population repertoire. Pooled playbacks consisted of 10 different 30 sec segments of syllable bouts (Avisoft SASLab Pro; version 4.40). When sample size < 10 males, segment duration/individual was increased to reach 310 sec of total playback length (e.g., in *M*.*m*.*d*.: four 76.5 seconds segments of uttered syllable bouts due to a low number of vocalizers (N = 4); Hybrid 1 & 2 were merged as test population to reach N = 10). Pooled USV playbacks from 4–10 males were used as a composite to control for individual variation in male USVs [8]. We conducted an experiment to investigate female responses to the pooled versus non-pooled playbacks to determine whether there is anything unusual about how females react to pooling individuals males. Females (*M*.*m*.*m*.1; N = 6, 184 ± 56.2 SD d of age at trial) could choose between a playback of pooled USVs of 10 males (*M*.*m*.*m*.1) versus a playback of an individual male USV (*M*.*m*.*m*.1, five 61 s repetitions of uttered syllable bouts). Females spent significantly more time at the fence in front of the pooled USV playback versus an individual male USV, but no further preference was found (Wilcoxon signed-rank, Median [sec] (IQR): Fence: 19.5 (7.1–67.4) vs. 7.87 (5.6–24.6), Z = -2.023, p = 0.043; Fence + Speaker zone: 31.3 (7.4–112.6) vs. 33.3 (18.1–121.1), Z = -0.105, p = 0.917; Fence + Speaker + Middle zone: 43.9 (14.5–127.7) vs. 40.8 (23.1–131.4), Z = -0.943, p = 0.345) (S2B Fig). Thus, our findings indicate that there is a positive or no effect of pooling individual male USVs to test female preferences, which means that pooling does not explain our negative results.

To examine female response to male USVs from different populations and species, estrous female mice (*M*.*m*.*m*.1; N = 20; 172.2 ± 11.7 day of age on the first use in the experiment) were tested for their response to pooled USV playbacks of males of their own population (*M*.*m*.*m*.1) compared to playbacks of a population with which they had not previously interacted (*M*.*m*.*m*.2, *M*.*m*.*m*.3, *M*.*m*.*d*., Hybrids and *M*.*s*. males) (Table 1). Here, we define a trial as a single test of a female’s response to two different populations and/or species. Females were tested once per trial in 5 different trials. Each trial took between 2–6 days to complete depending upon females’ estrus status. Time between trials varied between 2–3 weeks. The order of trials was the same for all females (*M*.*m*.*d*., *M*.*m*.*m*.2, *M*.*m*.*m*.3, *M*.*s*., Hybrids). For each trial a new pooled USV playback of their own population was composed. The side of playback presentation was balanced within subjects and reversed in subsequent trials.

**Table 1:** Experimental design of inter- and intraspecific playback experiments

Comparison	USV Playback Side 1	USV Playback Side 2
**Interspecific**		
*M*.*m*.*m*. vs. *M*.*s*.	*M*.*m*.*m*.1	*M*.*s*.
**Intersubspecific**		
*M*.*m*.*m*. vs. *M*.*m*.*d*.	*M*.*m*.*m*.1	*M*.*m*.*d*.
*M*.*m*.*m*. vs. *M*.*m*.*m*. x *M*.*m*.*d*.	*M*.*m*.*m*.1	Hybrids
**Intraspecific**		
*M*.*m*.*m*. vs. *M*.*m*.*m*.	*M*.*m*.*m*.1	*M*.*m*.*m*.2
	*M*.*m*.*m*.1	*M*.*m*.*m*.3

#### Data analyses and statistical tests

All behavioral trials were checked for a general side bias using nonparametric Wilcoxon signed-rank tests. The initial preference (side of first entry), latency of first entry, and the number of visits in each arm were also recorded. Initial preferences were tested for significance using a binomial test with an expected proportion of 0.5 (50%). For comparisons of latency of first entry the time for the not chosen arm was set to 310 s (total time of a trial). When the means of retention times in proximity zones were non-normally distributed, Wilcoxon signed-rank tests were applied. The time females spent sniffing at urine stimuli was determined with repeated measures ANOVAs with ‘male USV’ as the between subject factor and female as covariate. Sniffing behavior at the enhancing odor stimuli did not differ between cues (population-pools) and sides of the experimental apparatus indicating that these olfactory stimuli in general do not affect timed USV preferences (data not shown).

Female responses were further analyzed with respect to playback call composition. Although playbacks were standardized by the total number of syllables, ‘Frequency-Step’ syllables appeared irregularly in male vocalizations. The effect of individual variation in the numbers of ‘Frequency-Step’ syllables within playbacks on female behavior was tested using linear mixed models for repeated measures with the individual number of ‘Frequency-Step’ syllables as a fixed effect and trial number as a random effect.

### Ethics

The study was approved by the institutional ethics committee of the University of Veterinary Medicine, Vienna, in accordance with Good Scientific Practice guidelines and national legislation. Mice were trapped on private land with the permission of the owners. Trapping of the founder individuals was conducted overnight with Sherman live traps. Each trap was set with food and nesting materials. Traps were checked twice during the night for occupancy and trapped individuals were immediately removed and placed individually into standard mouse cages (type II). Trapping was performed in accordance with national legislation and approved by the MA 22 (Municipality for Environment and Conservation of Vienna).

## Results

### Experiment 1—USV variability among populations and species of male mice

#### Vocalizing behavior

Not all males emitted USVs when presented with female urine, and significant differences in the proportion of vocalizing versus non-vocalizing individuals were found between different *Mus* species (range: 29–87% vocalizing males; N = 127, χ² = 33.021, df = 4, p < 0.001). Due to differences in variance of in the proportion of vocalizing males (Levene tests for equality of variance: N = 114, F_6,107_ = 5.368, p < 0.001), *M*.*m*. populations could only partially be merged (*M*.*m*.*m*.1 & *M*.*m*.*m*.4; *M*.*m*.*m*.2 & *M*.*m*.*m*.3, Hybrid 1 & 2) resulting in a total of 5 groups for the species comparison. The proportion of vocalizing males also differed among *M*. *m*. subspecies (N = 114, χ² = 29.537m df = 3, p < 0.001) with *M*.*m*.*d*. showing the lowest proportion of vocalizing males (29%) and among *M*.*m*.*m*. populations (range: 31–87% vocalizing males; N = 78, χ² = 19.544, df = 3, p < 0.001).

#### Latency to vocalize

**Fig 2:**
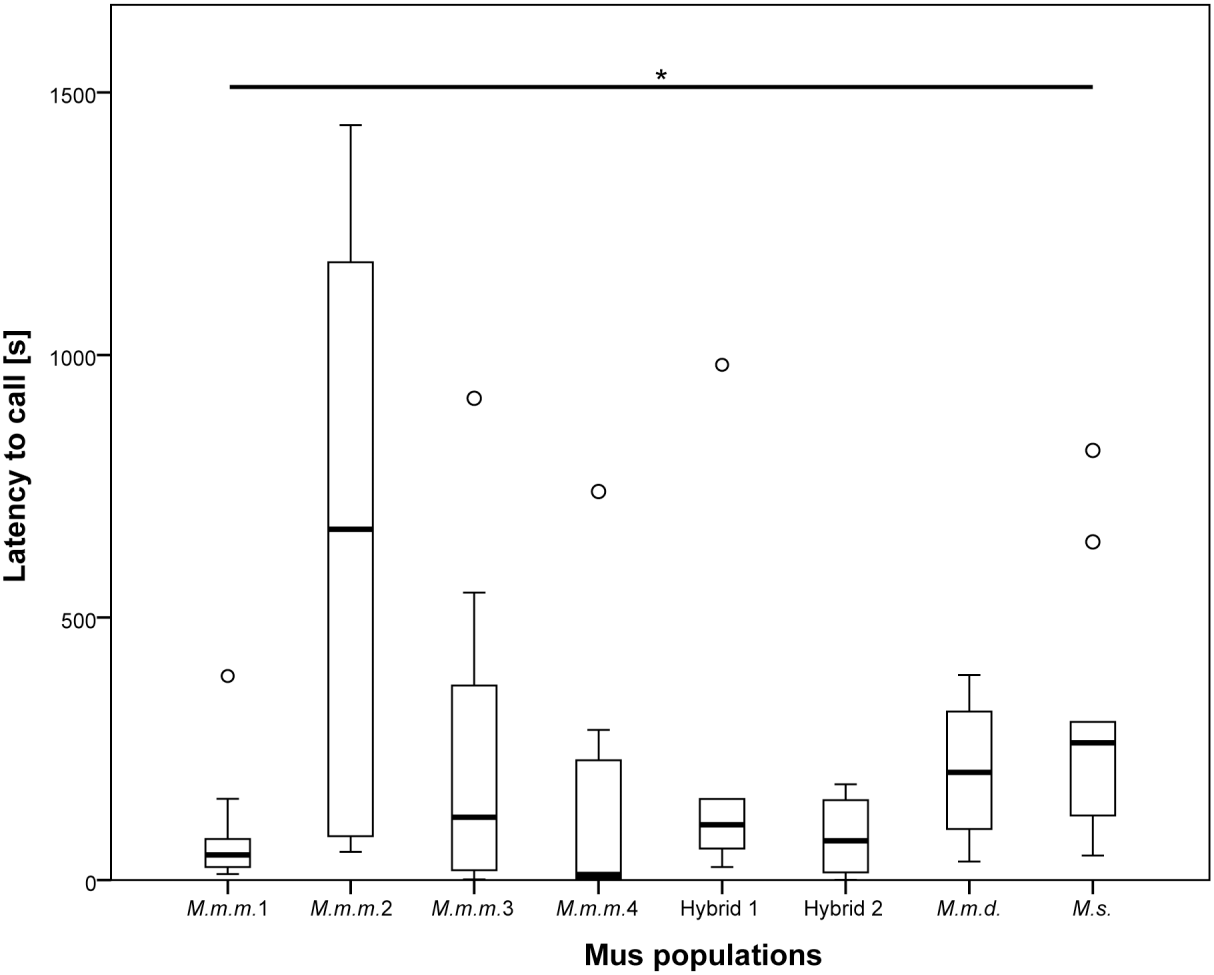
Latency to vocalize of males of different *Mus* populations. N = 10, except for Hybrid 1 = 5, Hybrid 2 = 7 and *M*.*m*.*d*. = 4. Significant differences were observed among populations (p = 0.026). The box represents the interquartile range that contains the middle 50% of values. The thick line across the box indicates the median. Upper and lower whiskers limits are set to 1.5 x interquartile range above and below the third and first quartile. Outliers shown as circles. The asterisk represents significance at a level of p < 0.05.

We found large differences among vocalizing males in their latency to first vocalization (0.5 to 1437.5 sec) with significant variation among *M*.*m*.*m*. populations (Median (s): 10.82–667.90; Kruskall—Wallis test: N = 40, χ² = 10.64, df = 3, p = 0.014; Levene test of equality of variance: N = 40; F3,36 = 9,720; p ≤ 0.0001), preventing the combined analysis of the *M*.*m*.*m*. populations for further statistical analyses. No significant difference was found in latency to vocalize when comparing *Mus* species (*M*.*m*.*vs M*.*s*.) or *M*.*m*. subspecies (*M*.*m*.*d*. *vs*. *Hybrids*) when *M*.*m*.*m*. was excluded (Mann-Whitney U test: Species: N = 26, U = 48.0, p = 0.097; Subspecies: N = 16, U = 13.0, p = 0.182), but significant differences were observed when including *M*.*m*.*m*. as separate taxa in the analysis (Kruskall—Wallis test: N = 66, χ² = 15.94, df = 7, p = 0.026), results which were supported by post hoc Mann-Whitney U tests (Fig 2). Significant differences in latency to vocalize were found in the following comparisons: *M*.*s*. vs. *M*.*m*.*m*.4: N = 20, U = 23.0, p = 0.041; *M*.*s*. vs. *M*.*m*.*m*.1: N = 20, U = 17.0, p = 0.013; *M*.*s*. vs. Hybrid 2: N = 17, U = 14.0, p = 0.04; *M*.*m*.*m*.2 vs. *M*.*m*.*m*.4: N = 20, U = 17.0, p = 0.013; *M*.*m*.*m*.1 vs. *M*.*m*.*m*.2: N = 20, U = 11, p = 0.003; *M*.*m*.*m*.2 vs. Hybrid 2: N = 17, U = 13, p = 0.032) (Mann- Whitney U tests; all p-values significant after FDR).

#### Quantity and quality of USVs

Among vocalizing males, individuals uttered 31–2661 syllables during the 30 min recording period with a mean syllable count per population from 256 ± 347.4 SD to 1057 ± 1023.8 SD. *M*.*m*. populations and hybrid lines were pooled for interspecific comparison of syllable rate because intra—group variance was low (Levene test of equality of variance: N = 56, F_6-49_ = 1.940, p = 0.093). No significant differences in USV emission rate were observed among species (Mann-Whitney U test: N = 66, U = 270.0, p = 0.858) or populations (Kruskal—Wallis test: N = 66, χ ² = 12.286, df = 7, p = 0.092).

Age of males did not have an effect on latency to vocalize or on USVs emission rate among all populations and species (Latency: Spearman rank correlation: N = 66, rs = 0.024, p = 0.849; Number of syllables: Spearman rank correlation: N = 66, rs = - 0.025, p = 0.843) (data not shown).

**Fig 3:**
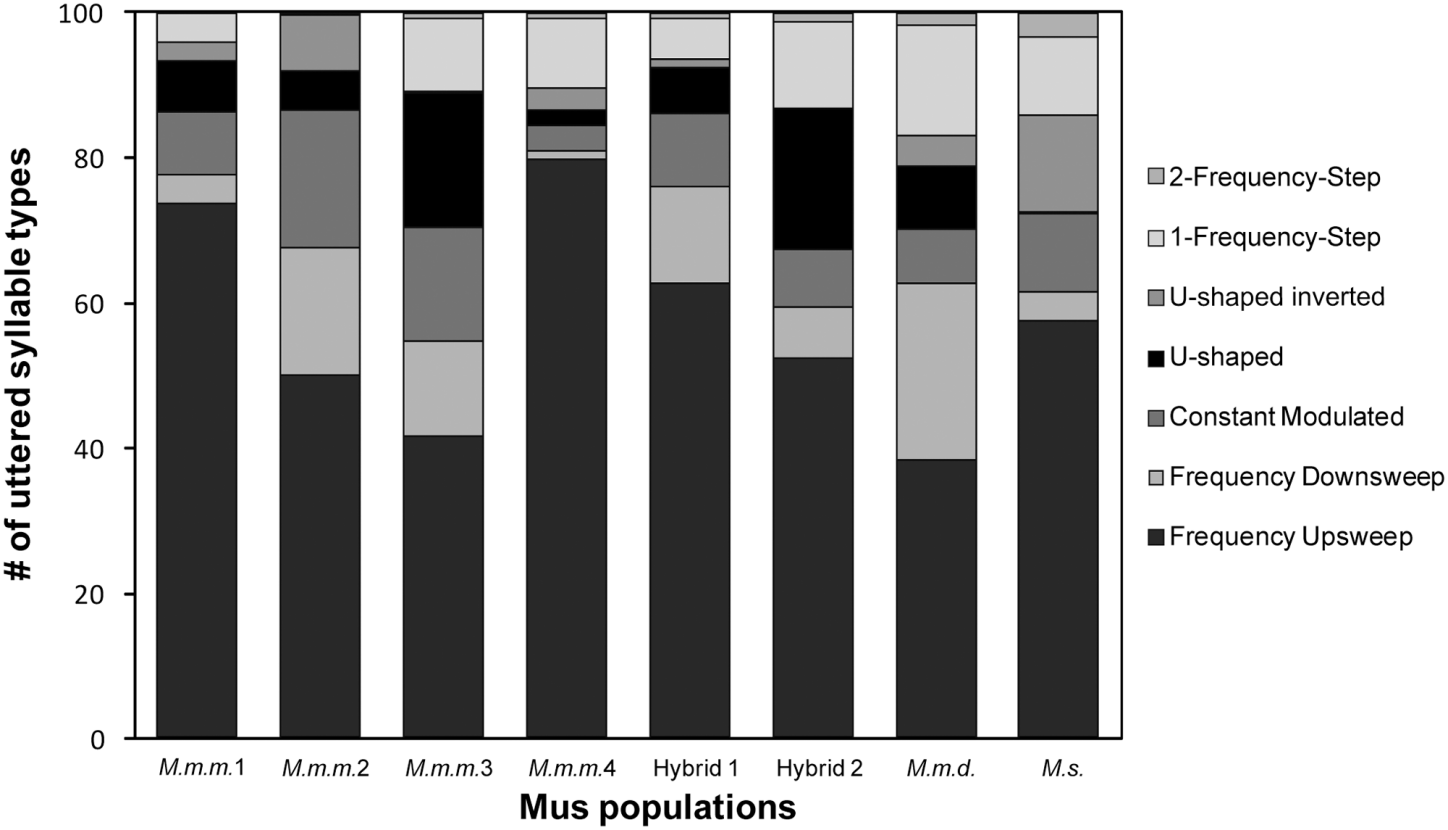
USV repertoires of different *Mus* populations based on the first 100 syllables. N = 10, except for Hybrid 1 = 5, Hybrid 2 = 7 and *M*.*m*.*d*. = 4. Syllable type ‘Frequency Upsweep’ is the most common type with a proportion of on average 56.6% (± 21.35 SD) of the repertoire. Syllable types ‘U-shaped’, ‘U-shaped Inverted’ and ‘2-Frequency Step’ were not emitted by all populations.

Populations differed in the proportion of different syllable types produced (Fig 3). ‘Frequency Upsweep’ (56.6% ± 21.4 SD) was the most common syllable type among all populations and ‘2-Frequency-Step’ and ‘U-Shaped Inverted’ syllables were not present in all populations. When analyzing the proportion of syllable types, significant differences were observed across populations (Kruskall—Wallis test: N = 66, df = 7: 'Frequency Upsweep': χ² = 34.722, p < 0.001; 'Frequency Downsweep': χ² = 35.606, p < 0.001; ‘Constant Modulated’: χ² = 20.447, p = 0.005; ‘U-Shaped’: χ² = 40.913, p < 0.001; ‘U-Shaped Inverted’: χ² = 41.919, p < 0.001; ‘2-Frequency-Step’: χ² = 20.414, p = 0.004; S1 Table). Syllable type ‘1-Frequency-Step’ did not differ significantly among populations (χ² = 10.971, p = 0.140).

#### Spectral and temporal characteristics of USVs

Spectral and temporal characteristic data were reduced to 6 variables (5 PC components and duration). Two extracted frequency PC components explained 89% of the variation in the 12-frequency variables in the data set (Table 2). The amplitude PC component explained 83% of the variation in the 4-amplitude variables in the data set (Table 2). The bandwidth PC component explained 42% of variation in the 4-bandwidth variables, while the entropy PC component explained 66% of variation in the 4-entropy variables in the data set (Table 2).

**Table 2:** Axis loadings from principal components analysis of 25 spectral and temporal characteristics of USVs averaged for each individual across all call types. The major contribution to the PC axis is explained under each PC axis. Component loadings are presented.

	PC1 Freq	PC2 Freq	PC AMP	PC Band	PC ENT
% Variation	79.47%	9.34%	83.19%	42.31%	65.59%
**pFstart**	0.84	0.53			
**pFend**	0.87	-0.28			
**minFstart**	0.85	0.52			
**maxFstart**	0.85	0.52			
**minFend**	0.88	-0.28			
**maxFend**	0.87	-0.28			
**pFcenter**	0.88	-0.12			
**minFcenter**	0.90	-0.11			
**maxFcenter**	0.88	0.07			
**pFmax**	0.96	-0.11			
**minFmax**	0.96	-0.11			
**maxFmax**	0.96	-0.11			
**pAMPstart**			0.93		
**pAMPend**			0.95		
**pAMPcenter**			0.79		
**pAMPmax**			0.97		
**bandstart**				0.79	
**bandend**				0.56	
**bandcenter**				0.49	
**bandmax**				0.72	
**entstart**					0.76
**entend**					0.85
**entcenter**					0.80
**entmax**					0.84

Abbreviations: p = peak, F = frequency, AMP = amplitude, band = bandwidth, ent = entropy.

**Fig 4:**
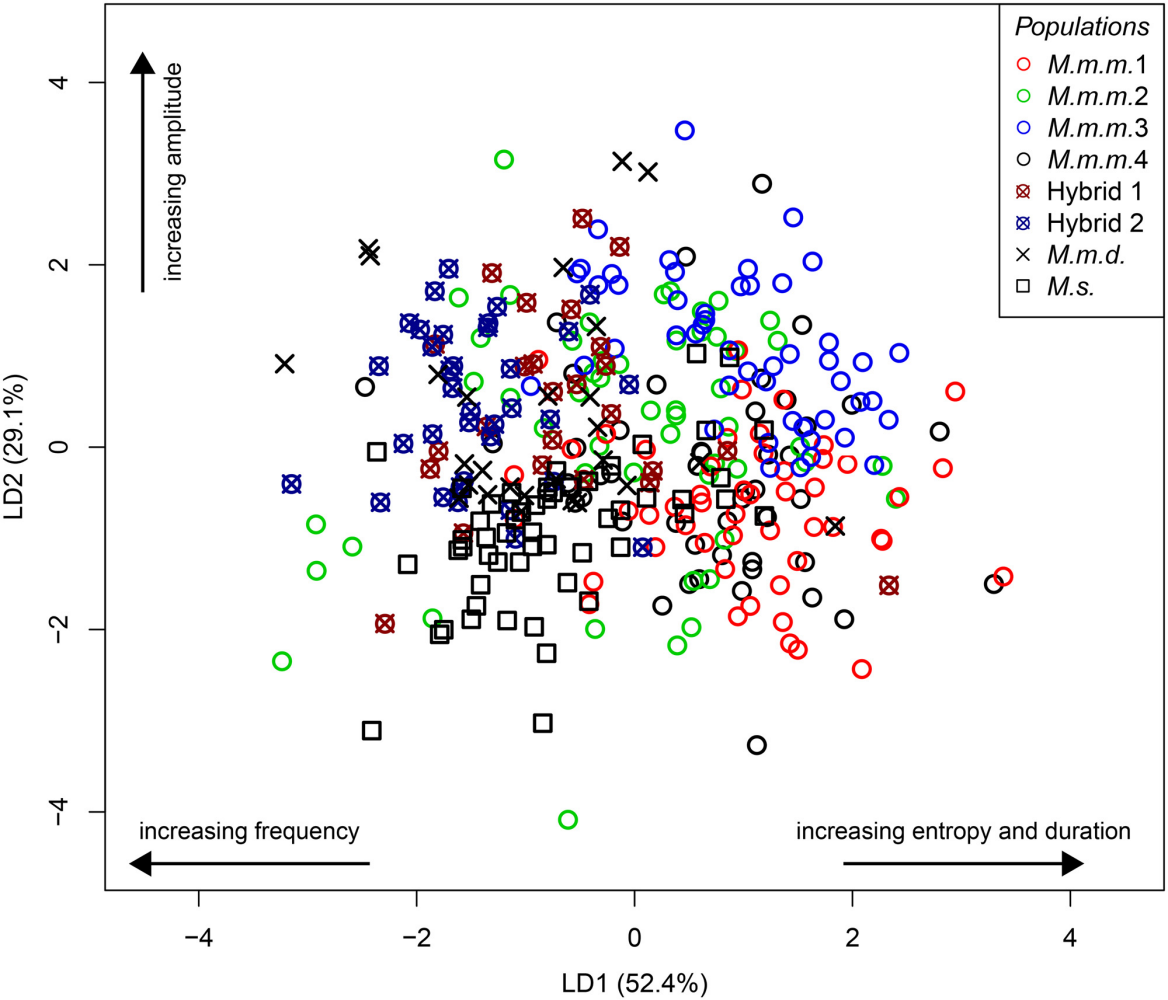
Classification plot of the first two linear discriminant axes for DFA on reduced data set (6 PC variables and duration) to classify all 8 populations. For interpretation, the proportion of the data set explained by each axis is given and the standardized co-efficient with the highest absolute values are presented.

SVM exhibited higher discriminating efficiency at the interspecific (Table 3) and intersubspecific level (Table 4), but DFA classified vocalizations to intraspecific populations more consistently than SVM (Table 5). As expected, both SVMs and DFAs performed better when classifying higher order taxonomic groups (interspecific and intersubspecific levels; Tables 3 and 4) than when classifying populations within a subspecies (Table 5). Across all 8 populations considered, both SVM and DFA had classification rates lower than 46% (43.12% and 45.8% respectively) because of considerable overlap among all *M*.*m*. populations and overlap between *M*.*m*. and *M*.*s*. populations (Fig 4).

**Table 3:** Mann-Whitney U results for comparisons between *Mus musculus (M*.*m*.*)* and *M*. *spicilegus* (*M*.*s*.) using all USV types combined and comparing within USV types. All variables are shown for the combined analysis but only significant variables shown for the within call-type analysis.

	# Individuals						
	*M*.*m*.	*M*.*s*.	U	Standardized test statistic	P	SVM Classification	DFA Classification
**All USV types combined**	**289**	**54**				89.21%	86.90%
Duration			6020.00	-2.66	0.01		
PC ENT			5321.00	-3.71	0.00		
PC BAND			6827.00	-1.46	0.14		
PC AMP			3517.00	-6.41	<0.001		
PC F1			10161.00	3.53	<0.001		
PC F2			8022.00	0.33	0.74		
**Frequency Upsweep**	**56**	**10**					
Duration			136.50	-2.57	0.01		
PC AMP			72.00	-3.72	<0.001		
PC F1			419.00	2.49	0.013		
PC F2			459.00	3.20	<0.001		
**Frequency Downsweep**	**50**	**9**					
PC AMP			98.00	-2.68	0.007		
PC F2			121.00	-2.19	0.03		
**Constant Modulated**	**56**	**10**					
PC AMP			95.00	-3.31	0.00		
PC F1			397.00	2.09	0.04		
**U-Shaped**	**52**	**2**	sample size too small for comparison				
**U-Shaped Inverted**	**33**	**10**					
PC ENT			88.00	-2.21	0.026		
PC AMP			67.00	-2.81	0.004		
PC F1			251.00	2.47	0.012		
PC F2			234.00	1.98	0.048		
**1-Frequency-Step**	**31**	**8**					
PC AMP			57.00	-2.33	0.02		
**2-Frequency-Step**	**11**	**5**	sample size too small for comparison				

**Table 4:** Mann-Whitney U results for comparisons between *Mus musculus musculus* (*M*.*m*.*m*.) and *Mus musculus domesticus* (*M*.*m*.*d*.) using all USV types combined and comparing within USV types. All variables are shown for the combined analysis but only significant variables shown for the within call-type analysis.

	# Indiviudals						
	*M*.*m*.*d*.	*M*.*m*.*m*.	U	Standardized test statistic	P	SVM Classification	DFA Classification
**All USVs types combined**	**27**	**201**				88.04%	79.4%
Duration			3555.00	2.62	0.09		
PC ENT			3598.00	2.75	0.01		
PC BAND			2126.00	-1.83	0.07		
PC AMP			2552.00	-0.60	0.55		
PC F1			1689.00	-3.18	0.001		
PC F2			1499.00	-43.77	<0.001		
**Frequency Upsweep**	**4**	**40**	sample size too small for comparison				
**Frequency Downsweep**	**4**	**34**	sample size too small for comparison				
**Constant Modulated**	**4**	**40**	sample size too small for comparison				
**U-Shaped**	**4**	**36**	sample size too small for comparison				
**U-Shaped Inverted**	**4**	**28**	sample size too small for comparison				
**1-Frequency-Step**	**4**	**19**	sample size too small for comparison				
**2-Frequency-Step**	**3**	**4**	sample size too small for comparison				

**Table 5:** Kruskal-Wallis results for comparisons among the four *Mus musculus musculus* populations using all USV types combined and comparing within USV types. All variables are shown for the combined analysis but only significant variables shown for the within call-type analysis.

	# Individuals								
	*M*.*m*.*m*.1	*M*.*m*.*m*.2	*M*.*m*.*m*.3	*M*.*m*.*m*.4	Test Statistic	Degrees of Freedom	P	SVM Classification	DFA Classification
**All USVs combined**	**52**	**52**	**49**	**48**				41.79%	52.20%
Duration					10.48	3	0.02		
PC ENT					38.48	3	<0.001		
PC BAND					9.16	3	0.02		
PC AMP					26.72	3	<0.001		
PC F1					21.63	3	<0.001		
PC F2					19.70	3	<0.001		
**Frequency Upsweep**	**10**	**10**	**10**	**10**					
PC ENT					12.04	3	0.01		
PC F2					18.73	3	<0.001		
**Frequency Downsweep**	**10**	**10**	**10**	**4**					
PC AMP					10.24	3	0.02		
**Constant Modulated**	**10**	**10**	**10**	**10**					
Duration					8.99	3	0.03		
PC ENT					7.90	3	0.05		
PC AMP					11.69	3	0.01		
PC F1					9.23	3	0.03		
PC F2					10.61	3	0.01		
**U-shaped**	**9**	**10**	**10**	**7**					
PC AMP					8.37	3	0.04		
**U-shaped Inverted**	**7**	**10**	**3**	**8**	no significant differences				
**1-Frequency-Step**	**6**	**2**	**5**	**6**	sample size too small for comparison				
**2 Frequency-Step**	**0**	**0**	**1**	**3**	sample size too small for comparison				

We found striking variation in the USVs of different males (Fig 4), and although there was much overlap between different subspecies and populations, *M*.*m*. USVs differed from those of *M*.*s*. when considering all USVs syllable types, indicating species differences in spectral and temporal characteristics USVs (Table 3). Specifically, the parameters that differed most significantly between *Mus* species were duration and the PC axes describing frequency, amplitude, and entropy (Table 3). These differences are reflected in the separation of *M*.*s*. from the other population types in Fig 4. *M*.*s*. vocalized at a higher frequency (74.69 ± 1.01kHz vs. 69.75 ± 0.61 kHz peak frequency at maximum amplitude), lower amplitude (-22.59 ± 0.50 vs. -18.0± 0.40 dB peak amplitude) and entropy than *M*.*m*. (0.199 ± 0.001 vs. 0.187 ± 0.001; Table 3, see Fig 4). In addition, *M*.*s*. USVs were shorter in duration than *M*.*m*. USVs (0.030 ± 0.001 vs. 0.0325± 0.001 sec). These species differences, especially regarding frequency and amplitude were generally consistent across USV syllable types (Table 3). Both SVM and DFA classification approaches were effective at classifying over 85% of USVs to the species level (Table 3).

Intraspecific comparisons between subspecies (*M*.*m*.*m*. and *M*.*m*.*d*.; Table 4) and within *M*.*m*.*m*. populations (Table 5) show differences in USVs based on spectral characters. However, SVM and DFA classification were much more effective at discriminating *M*.*m*. subspecies compared to populations within *M*.*m*.*m*. While both SVM and DFA classification were equally effective at discriminating two different subspecies (over 79%; Table 4) as for classifying two species (over 86%; Table 4), discriminating mice from different populations within *M*.*m*.*m*. was less than 42% for SVM and less than 53% for DFA (Table 5). Sample sizes were generally too small to examine intrasubspecific variation within particular USV syllable types (Table 4) as a result of the *M*.*m*.*d*. population consisting of only 4 individuals. However, intraspecific comparisons among *M*.*m*.*m*. populations were possible for ‘Constant modulated’, ‘Frequency upsweep’, and ‘U shaped’ syllable types with both amplitude and frequency differing among populations (Table 5).

### Experiment 2 –Female discrimination of male USV playbacks

**Fig 5:**
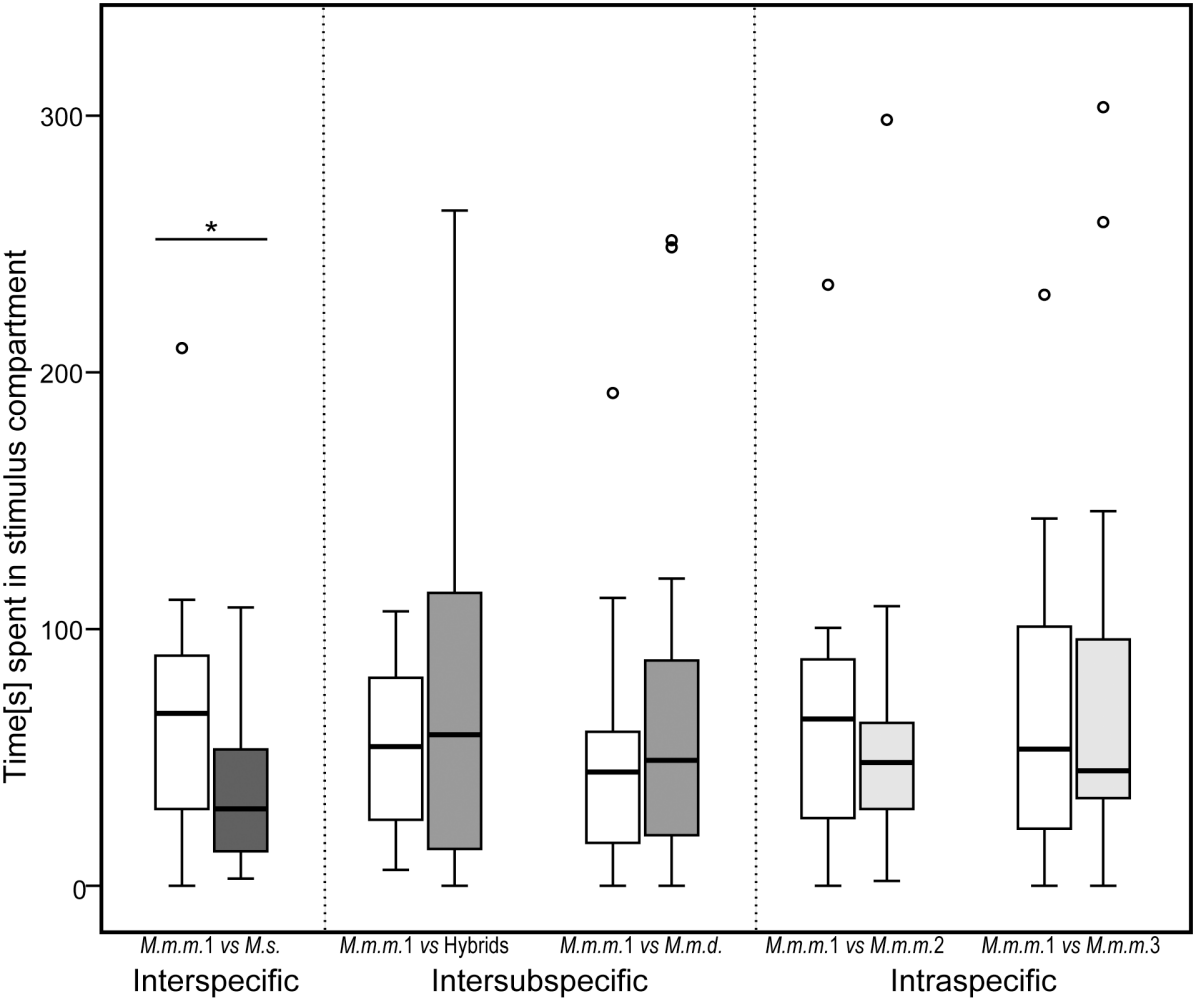
Female preferences for male USV playbacks. Time (s) females (N = 20 except trial 2: *M*.*m*.*m*.1 *vs M*.*m*.*m*.2, N = 19) spent at fence / speaker zone in proximity to male USV playbacks. The box represents the interquartile range that contains the middle 50% of values. The thick line across the box indicates the median. Upper and lower whiskers limits are set to 1.5 x interquartile range above and below the third and first quartile. Outliers shown as circles. The asterisk represents significance at a level of p < 0.05.

When presented with USV playbacks of males from their own population (*M*.*m*.*m*.1) versus a different species (*M*.*s*.), females (N = 20) spent significantly more time at the fence and in the zones next to conspecific male USV (Fig 5, Table 6). Females also spent more time self-grooming in zones (Fence + Speaker) in closer proximity to the playbacks of USVs from conspecific (*M*.*m*.*m*.1) than to heterospecific males: median (IQR) (%): 0 (0–36) vs. 0 (0–0), Z = -2.497, p = 0.013. Females spent an equal amount of time near playbacks of *M*.*m*.*d*. and hybrid males compared to their own subspecies (Fig 5, Table 6). Similarly, females did not discriminate between USV playbacks of males from different *M*.*m*.*m*. populations (Table 6), nor did they show differences in grooming behavior (data not shown). Also, we found no other differences in female responses to USVs from different populations in initial preference, latency to enter one stimulus compartment, or number of visits (data not shown).

**Table 6:** Median time [sec] (IQR) *M*.*m*.*m*.1 females spent in designated areas next to respective USV playback. Wilcoxon signed rank test indicated significant differences (in bold) when comparing female preferences at the interspecific level.

	Interspecific				Intersubspecific								Intraspecific							
Playback	*M*.*m*.*m*.1	*M*.*s*.	Z	P	*M*.*m*.*m*.1	*M*.*m*.*d*	Z	P	*M*.*m*.*m*.1	*M*.*m*.*m*. x *M*.*m*.*d*.	Z	P	*M*.*m*.*m*.1	*M*.*m*.*m*.2	Z	p	*M*.*m*.*m*.1	*M*.*m*.*m*.3	Z	p
**Fence**	31.0 (18.1–50.1)	19.0 (6.5–30.9)	-2.415	**0.016**	22.8 (10.3–35.6)	16.7 (7.3–41.7)	- 0.448	0.654	28.7 (12.1–47.0)	27.5 (7.1–48.0)	-1.045	0.296	34.4 (17.1–51.8)	22.4 (14.7–31.8)	- 0.684	0.494	21.9 (8.3–31.7)	25.0 (16.5–40.3)	-0187	0.852
**Fence + Speaker zone**	67.1 (28.7–93.1)	30.0 (12.6–53.7)	-1.979	**0.048**	44.3 (14.7–62.3)	48.8 (17.7–90.8)	- 0.402	0.687	54.2 (22.5–81.9)	58.8 (12.0–123.9)	-0.075	0.940	65.0 (23.0–89.3)	48.0 (29.3–66.3)	-1.502	0.133	53.3 (22.1–110.4)	44.8 (33.0–100.7)	-0.224	0.823
**Fence + Speaker + Middle zone**	74.9 (47.6–122.2)	37.5 (14.0–81.7)	-2.277	**0.023**	57.5 (21.1–84.4)	53.7 (27.7–113.0)	- 0.859	0.391	70.7 (28.8–94.7)	66.1 (24.4–147.0)	-1.045	0.296	78.1 (31.2–97.1)	62.3 (33.0–78.9)	- 0.282	0.778	69.6 (34.3–130.5)	64.8 (40.8–113.7)	- 0.000	1.000

Proportions of ‘Frequency-Step’ syllables of individual males within the playbacks (0.8–20.8%) did not influence female preference (*M*.*m*.*m*.1 vs. *M*.*m*.*m*.2: Linear mixed model: F = 0.487, p = 0.494; *M*.*m*.*m*.1 vs. *M*.*m*.*m*.3: Linear mixed model: F = 0.075, p = 0.785; *M*.*m*.*m*.1 vs. *M*.*m*.*d*.: Linear mixed model: F = 1,033, p = 0.322; *M*.*m*.*m*.1 vs. Hybrids: Linear mixed model: F = 1.591, p = 0.222; *M*.*m*.*m*.1 vs. *M*.*s*.: Linear mixed model: F = 3.695, p = 0.070).

## Discussion

Our analysis revealed significant differences in several features of male USVs between *M*. *m*. *musculus* and *M*. *spicilegus*, including latency to vocalize, syllable repertoire, spectral and temporal characteristics. We also found that female *M*. *m*. *musculus* were able to distinguish between the USVs of conspecific and heterospecific males, and spent significantly more time near calls from conspecific males. These results provide the first evidence that the USVs of males differ among *Mus* species and that females can discriminate and prefer the USVs of conspecific over heterospecific males. As playbacks were composed of recordings of a pool of individual males, rather than a single individual male on each side, these results cannot be explained by a simple preference for more dissimilar or less familiar calls due to females’ imprinting on paternal USVs [21]. Future studies are needed to test whether female preferences for the USVs of conspecific males are enhanced when combined with other stimuli from other sensory modalities (multimodal integration) [7, 61] and whether USVs are subject to sexual selection mediated by female choice. If male USVs mediate such inter-sexual interactions, then USVs would help to avoid genetically incompatible matings and provide a pre-zygotic reproductive isolation mechanism.

Females’ preferences for the USVs of conspecific males may be controlled by innate recognition or females may positively imprint on species-specific features of vocalizations of males in their family—as found in birds [62]. The innate preference of female laboratory mice for the USVs of males from a different strain has been shown to disappear when they are reared without fathers, emphasizing the necessity of close exposure to USV [21] and disassortative preferences can be reversed by cross-fostering [21], suggesting that female preferences for male USVs are controlled by classical (paternal) imprinting, and that females *negatively* imprint on the USVs of males in their own family. If females *positively* imprint on species-specific features of USVs from males in their family, then such dual imprinting may allow females to avoid the extremes of inter-specific hybridization and close inbreeding (optimal outbreeding) [63].

We compared USVs of male *M*. *musculus* on an intraspecific level, and we found significant differences between two subspecies (*musculus* and *domesticus)* and also between hybrids and their parental subspecies. The classification of these groups of mice in our models (both SVM and DFA) was relatively high and comparable to classification levels at the species level. Yet, despite these differences in male USVs, we found no evidence that females discriminated between the playbacks of male USVs of *M*. *musculus* subspecies (*M*.*m*.*m*. versus *M*.*m*.*d*.) or between conspecific males versus hybrids of these subspecies. Thus, the lack of female *M*. *m*. *musculus* discrimination does not appear to be due to a lack of differences in male USVs between subspecies. Alternatively, pooled USV playbacks might hamper female discrimination, as they are more variable than natural calls and thereby might diffuse the subspecies signal. To rule out such potential experimental artifacts, female preferences should be tested with individual playbacks. Previous odor preference tests found that *M*. *m*. *musculus*, but not *M*. *m*. *domesticus*, mice from parapatric and sympatric populations showed significant assortative odor and partner preferences for their own subspecies [34, 40] but no assortative preference was found in allopatric populations [41]. House mice from the subspecies hybrid zone produce distinct urinary signals that are more pronounced compared to other areas (i. e. character displacement) [42]. The mice used in our study were collected far from the hybrid zone, and therefore, studies are needed to test whether females from sympatric populations show assortative preferences for subspecies male USVs.

We also found several features of male USVs that differ among *M*. *m*. *musculus* populations, but found no evidence that *M*. *m*. *musculus* females discriminate between USV playbacks of males at the population level, as predicted by the paternal imprinting hypothesis. Females are capable of distinguishing the USVs of siblings versus unrelated males (from within the same population), indicating that females can recognize subtle differences in male USVs [5]. As the males in our experiment were unrelated to the choosing females, and USV playback consisted of pools of several males, each USV population pool might have been perceived as equally distant at the intraspecific level. Nonetheless, our findings indicate that there are intraspecific differences in components of male USVs, especially within ‘Constant modulated’ and ‘Frequency upsweep’ syllable types, which likely exceed those found among laboratory mouse strains [26]. *M*. *musculus* mice in our study were reared in the same room, but mice from different populations were kept on separate racks. The hearing threshold of mice [64], and the high attenuation of ultrasound [65] makes it highly unlikely that mice were able to perceive USV from distances > 3m,. Nevertheless, it is possible that the lack of female discrimination for USVs of males from different *M*. *m*. *musculus* populations may be due to young females imprinting on the USVs of males from different cages and different racks. Female discrimination of individual males from differing populations on a small geographic scale is required to test the imprinting hypothesis. Also, further studies are needed to examine the acoustic preferences of females from different subspecies and populations. Female *M*. *m*. *domesticus* differ from *M*. *m*. *musculus* by being indiscriminant towards the urinary scent of males from their subspecies [34, 40, 66]. Therefore, females from other *M*. *m*. *musculus* populations, subspecies and species need to be tested with respect to their USV preferences.

Spectral features that differentiated the two species in our study were amplitude and frequency, whereas subspecies and populations mainly varied in duration and frequency, the latter two being consistent with differences among inbred mouse strains [49] and between wild and laboratory *Peromyscus* mice [25]. The calls of mound-building mice were quieter and of higher frequency than those of house mice. In addition, we also found evidence that variation in frequency, entropy, and to a lesser extent duration, discriminated species. Within *M*. *musculus* subspecies, frequency, duration, and entropy were important discriminating features. At population levels, we identified amplitude, frequency, entropy and duration as being important discriminatory parameters.

In addition to female preferences for conspecific male USVs (species recognition) and species differences in male USV characteristics, we found several results that should be considered in future studies on mouse USVs. First, our spectrographic analysis of USVs had a robust classification rate between species and subspecies and supported taxonomic differentiation between *M*. *m*. *musculus* and *M*. *m*. *domesticus*, but further research is needed to determine how well variation in USVs and which features coincide with phylogeny [67]. We used discriminant function analysis (DFA) and support vector machines (SVM) to discriminate species, subspecies, and populations based on the spectrographic features. In previous studies on bats, SVMs provided better classifications and accuracies than DFAs [68, 69]; however, in our study, both methods provided similar results. SVMs may not have outperformed DFAs because our data set, that needed to be divided into testing and training sets for the SVM, was small relative to previous studies. Regardless, both SVM and DFA approaches performed well when classifying *Mus* USVs at the species and subspecies level.

Second, we found significant variation in USVs among geographic populations of *M*. *m*. *musculus*, consistent with other evidence that male USVs are innate [20, 70] and influenced by genetic differences [8, 19, 49, 71, 72]. Syllable repertoire varied among *M*. *m*. *musculus* populations, as well as between *Mus* species. Geographic variation in song repertoire has been found in songbirds [73], bats [74] and wild singing mice (*Scotinomys*: [11]), which may also be due to genetic differences, as well as learning (‘dialects’) [75–77]. In our study, emission rate of 6 out of the 7 syllable types differed between populations with ‘Frequency upsweep’ being the most common syllable type in all populations (Fig 3). We found that latency to vocalize varied significantly between the *Mus* species and among *M*. *m*. *musculus* populations. Variation in latency and vocalization rate are known to differ among laboratory mouse strains [19, 49, 78–80], with dominant alleles coding for phenotypes with high vocalization rates and low latency [71]. Thus variation in USV production in our study may be influenced by genetic differences. Experimental populations derived from wild mice with the highest microsatellite diversity (*M*.*m*.*m*.1 and *M*.*m*.*m* 4.; [44]) had the highest proportion of vocalizing males (*M*.*m*.*m*.1: 87%; *M*.*m*.*m*.4.: 86%), shortest latencies to vocalize, and emitted the highest numbers of syllables. In contrast, populations with fewer vocalizing males (*M*.*m*.*m*.2: 39%; *M*.*m*.*m*.3: 31%; and *M*.*m*.*d*: 29%) emitted fewer USVs syllables with longer latencies. Interestingly, males remained consistent in their (non-) vocalizing behavior when tested repeatedly (data not shown), suggesting individual differences in sexual interest and motivation.

Finally, our results have implications for understanding whether male USV phenotypes provide reliable indicators of their quality or condition to rivals or potential mates. We found no evidence that female house mice prefer more complex ‘Frequency-Step’ syllables even though ‘Frequency-Step’ syllables are emitted by males at higher rates under conditions of sexual motivation from female scent [5]. We also found no evidence that male age (range 65–543 d) had any effect on latency to vocalize or vocalization rate. In rodents, serum testosterone concentration diminishes with age [81, 82], suggesting that androgen-mediated USVs should decrease with age [83], as does the expression of condition-dependent male secondary sexual traits in other species [84]. Future studies are clearly needed to examine which features of male USVs are attractive to females and whether they provide indicators of male quality or condition [7].

In summary, our study is the first to demonstrate significant variability in USVs among wild *Mus* species, subspecies, and *M*. *m*. *musculus* populations, and provides evidence that females can potentially use these signals to identify conspecifics. Thus, in addition to providing potential signatures of individual and kin recognition [5, 8], house mouse females may use male USVs for interspecific discrimination.

## Supporting Information

S1 Dataset Female preferences for male USV playback. (XLSX)

S2 Dataset Spectrographic mean data. (12 outliers removed).
(XLSX)

S1 Fig USV playback choice apparatus with the different zones indicated. Two enhancing urinary stimuli were presented in front of each speaker.
(TIF)

S2 Fig Female preferences for male USV playbacks controls. Time (s) females spent in zones (Fence + Speaker zone) in proximity to A) USV playback of a male call (N = 10) versus playback without USV and B) USV playback of a pool of male calls (N = 10) versus USV playback of 1 male. The box represents the interquartile range, the thick line indicated the medians and the whiskers include highest to lowest values (outliers excluded). Asterisks represent significance at a level of p ≤ 0.05.
(TIF)

S1 Table Intra-population comparison of individual syllables types. (Mann—Whitney-U tests, significant p—values after FDR control in bold).
(DOCX)
