# Supplementary figures and images for: Ultrasonic Vocalizations of Male Mice Differ among Species and Females Show Assortative Preferences for Male Calls
Source: PLoS One. 2015 Aug 26;10(8):e0134123. doi: 10.1371/journal.pone.0134123 (PMC4550448; doi:10.1371/journal.pone.0134123)

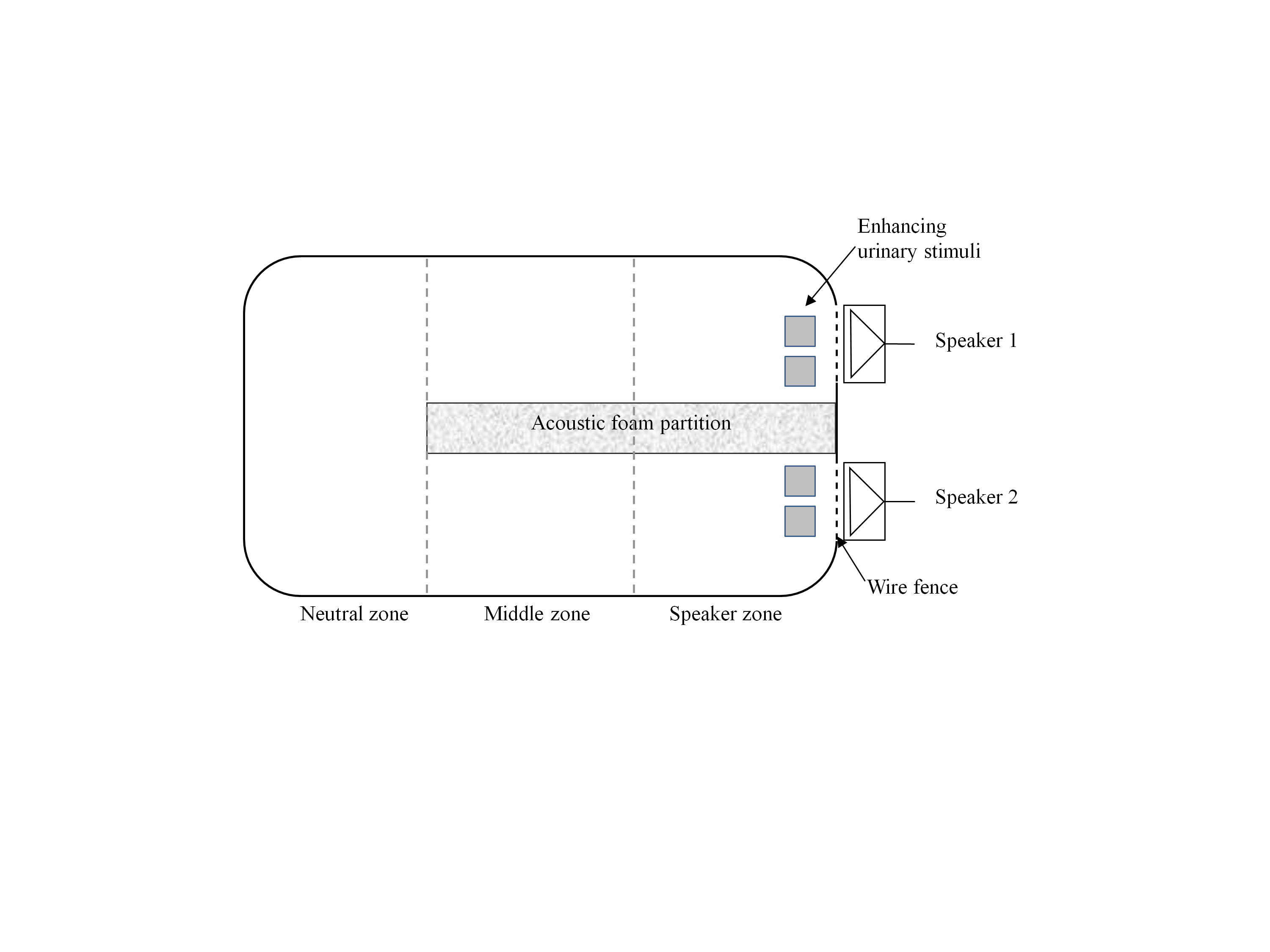

Supplement: S1 Fig — Two enhancing urinary stimuli were presented in front of each speaker. (TIF) [file pone.0134123.s003.tif]

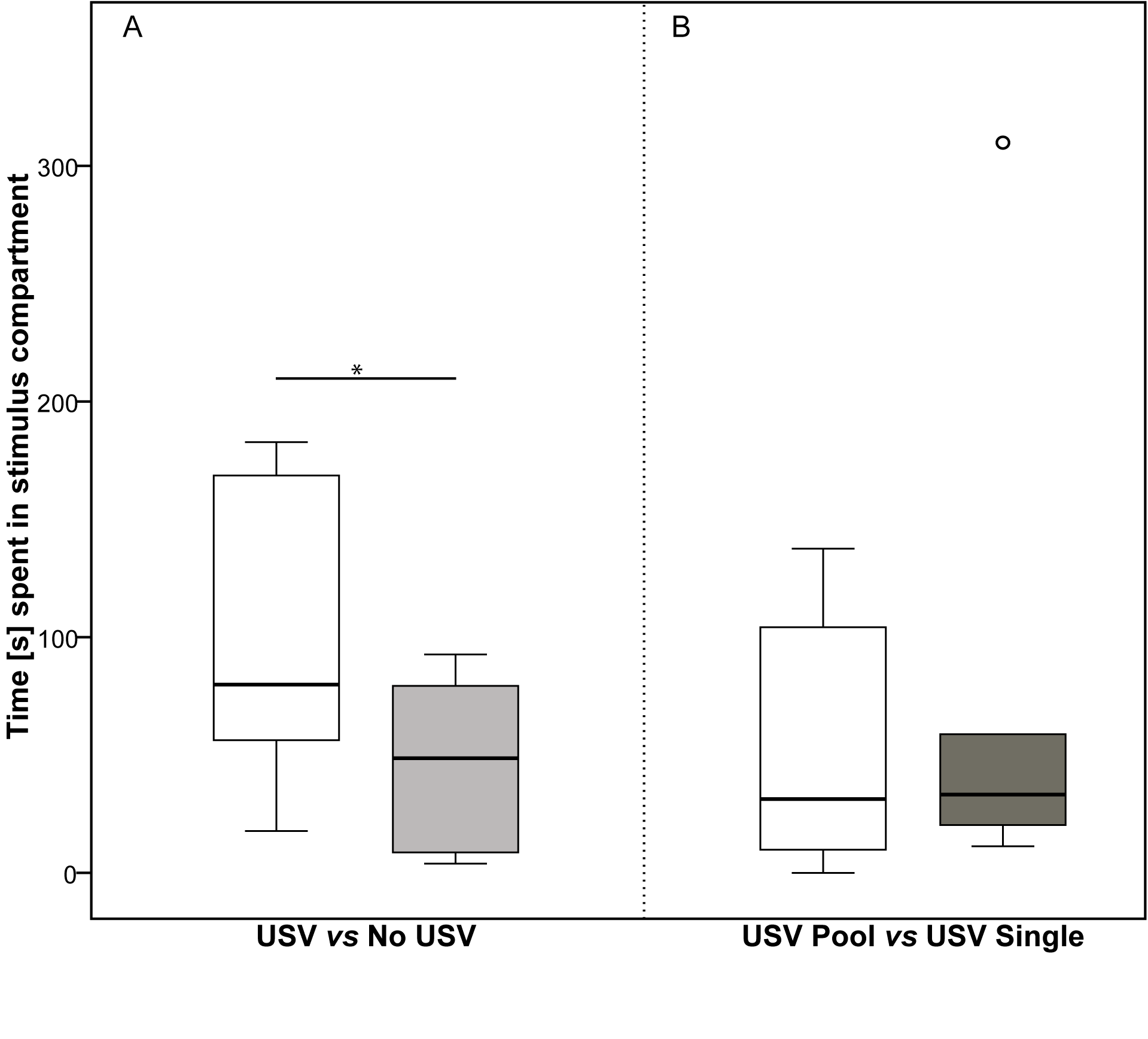

Supplement: S2 Fig — Time (s) females spent in zones (Fence + Speaker zone) in proximity to A) USV playback of a male call (N = 10) versus playback without USV and B) USV playback of a pool of male calls (N = 10) versus USV playback of 1 male. The box represents the interquartile range, the thick line indicated the medians and the whiskers include highest to lowest values (outliers excluded). Asterisks represent significance at a level of p ≤ 0.05. (TIF) [file pone.0134123.s004.tif]
