# Supplementary material for: Ultrasonic Vocalizations of Male Mice Differ among Species and Females Show Assortative Preferences for Male Calls
Source: PLoS One. 2015 Aug 26;10(8):e0134123. doi: 10.1371/journal.pone.0134123 (PMC4550448; doi:10.1371/journal.pone.0134123)
Supplement: S1 Table — (Mann—Whitney-U tests, significant p—values after FDR control in bold). (DOCX) [file pone.0134123.s005.docx]

# Supporting Information

S1 Table A-F: Intra-population comparison of individual syllables types, after significant inter-population differences (Mann–Whitney-U tests, significant p - values after FDR control in bold). Syllable type ‘1‑Frequency-Step’ (N = 517) did not differ significantly among populations.

Table A: ‘Frequency upsweep’ (N = 3740)

| **Frequency Upsweep** | ***M.m.m.1*** | ***M.m.m.2*** | ***M.m.m.3*** | ***M.m.m.4*** | **Hybrid 1** | **Hybrid 2** | ***M.m.d.*** |
| --- | --- | --- | --- | --- | --- | --- | --- |
| ***M.m.m.2*** | Z = -2.837  **p = 0.005** |  |  |  |  |  |  |
| ***M.m.m.3*** | Z = -3.569  **p≤0.001** | Z = -0.341  P = 0.733 |  |  |  |  |  |
| ***M.m.m.4*** | Z = -1.439  p = 0.150 | Z = -3.216  **p≤0.001** | Z = -3.483  **p≤0.001** |  |  |  |  |
| **Hybrid 1** | Z = -2.024  p = 0.043 | Z = -1.410  p = 0.159 | Z = -2.272  p = 0.023 | Z = -2.272  p = 0.023 |  |  |  |
| **Hybrid 2** | Z = -3.027  **p = 0.002** | Z = -0.342  p = 0.733 | Z = -0.879  p = 0.379 | Z = -2.931  **p = 0.003** | Z = -1.543  p = 0.123 |  |  |
| ***M.m.d.*** | Z = -2.832  **p = 0.005** | Z = -1.062  p = 0.288 | Z = -0.925  p = 0.355 | Z = -2.835  **p = 0.005** | Z = -2.449  **p = 0.014** | Z = -1.521  p = 0.131 |  |
| ***M.s.*** | Z = -2.195  **p = 0.029** | Z = -1.627  p = 0.105 | Z = -1.667  p = 0.105 | Z = -2.801  **p = 0.004** | Z = -0.491  p = 0.624 | Z = -0.782  p = 0.434 | Z = -2.409  **p = 0.016** |

Table B: ‘Frequency downsweep’ (N = 572)

| **Frequency downsweep** | ***M.m.m.1*** | ***M.m.m.2*** | ***M.m.m.3*** | ***M.m.m.4*** | **Hybrid 1** | **Hybrid 2** | ***M.m.d.*** |
| --- | --- | --- | --- | --- | --- | --- | --- |
| ***M.m.m.2*** | Z = -2.843  **p = 0.004** |  |  |  |  |  |  |
| ***M.m.m.3*** | Z = -1.863  p = 0.062 | Z = -0.493  p = 0.622 |  |  |  |  |  |
| ***M.m.m.4*** | Z = -2.579  **p = 0.01** | Z = -3.605  **p≤0.001** | Z = -3.185  **p = 0.001** |  |  |  |  |
| **Hybrid 1** | Z = -2.827  **p = 0.005** | Z = -1.858  p = 0.063 | Z = -1.323  p = 0.186 | Z = -3.165  **p = 0.002** |  |  |  |
| **Hybrid 2** | Z = -1.426  p = 0.154 | Z = -1.858  p = 0.063 | Z = -1.323  p = 0.186 | Z = -2.948  **p = 0.003** | Z = -2.126  p = 0.033 |  |  |
| ***M.m.d.*** | Z = -2.838  **p = 0.005** | Z = -1.277  p = 0.202 | Z = -1.346  p = 0.178 | Z = -2.944  **p = 0.003** | Z = -1.722  p = 0.085 | Z = -2.658  p **= 0.008** |  |
| ***M.s.*** | Z = -0.077  p = 0.939 | Z = -2.736  **p = 0.006** | Z = -1.903  p = 0.058 | Z = -2.621  **p = 0.009** | Z = -2.707  **p = 0.007** | Z = -1.278  p = 0.201 | Z = -2.841  **p = 0.004** |

Table C: ‘Constant modulated’ (N = 680)

| **Constant**  **modulated** | ***M.m.m.1*** | ***M.m.m.2*** | ***M.m.m.3*** | ***M.m.m.4*** | **Hybrid 1** | **Hybrid 2** | ***M.m.d.*** |
| --- | --- | --- | --- | --- | --- | --- | --- |
| ***M.m.m.2*** | Z=-2.843  **p=0.004** |  |  |  |  |  |  |
| ***M.m.m.3*** | Z=-1.289  p=0.197 | Z=-0.265  p=0.791 |  |  |  |  |  |
| ***M.m.m.4*** | Z=-2.964  **p=0.003** | Z=-3.191  **p=0.001** | Z=-2.820  **p=0.005** |  |  |  |  |
| **Hybrid 1** | Z=-0.619  p=0.536 | Z=-0.184  p=0.854 | Z=-0.368  p=0.713 | Z=-2.719  **p=0.007** |  |  |  |
| **Hybrid 2** | Z=-1.278  p=0.201 | Z=-1.858  p=0.063 | Z=-1.323  p=0.186 | Z=-1.585  p=0.113 | Z=-1.143  p=0.253 |  |  |
| ***M.m.d.*** | Z=-0.713  p=0.476 | Z=-1.628  p=0.103 | Z=-1.847  p=0.065 | Z=-0.791  p=0.429 | Z=-1.112  p=0.266 | Z=-0.572  p=0.567 |  |
| ***M.s.*** | Z=-0.456  p=0.648 | Z=-1.440  p=0.150 | Z=-1.250  p= 0.211 | Z=-2.739  p=0.006 | Z=-0.061  p=0.951 | Z=-1.028  p=0.304 | Z=-0.851  p=0.395 |

Table D: ‘U shaped’ (N = 506)

| **U shaped** | ***M.m.m.1*** | ***M.m.m.2*** | ***M.m.m.3*** | ***M.m.m.4*** | **Hybrid 1** | **Hybrid 2** | ***M.m.d.*** |
| --- | --- | --- | --- | --- | --- | --- | --- |
| ***M.m.m.2*** | Z=-0.308  p=0.758 |  |  |  |  |  |  |
| ***M.m.m.3*** | Z=-2.585  **p=0.010** | Z=-3.080  **p=0.002** |  |  |  |  |  |
| ***M.m.m.4*** | Z=-1.452  p=0.146 | Z=-1.912  P=0.056 | Z=-3.268  **p=0.001** |  |  |  |  |
| **Hybrid 1** | Z=-0.186  p=0.853 | Z=-0.560  p=0.575 | Z=-2.399  **p=0.016** | Z=-2.041  **p=0.041** |  |  |  |
| **Hybrid 2** | Z=-2.401  **p=0.016** | Z=-2.891  **p=0.004** | Z=-0.392  p=0.695 | Z=-3.332  **p=0.001** | Z=-2.030  **p=0.042** |  |  |
| ***M.m.d.*** | Z=-0.498  p=0.619 | Z=-0.857  p=0.391 | Z=-2.131  **p=0.033** | Z=-2.155  p=0.031 | Z=-0247  p=0.805 | Z=-1.701  p=0.089 |  |
| ***M.s.*** | Z=-3.277  **p=0.001** | Z=-3.704  **p=0.006** | Z=-3.839  **p≤0.001** | Z=-2.927  **p=0.003** | Z=-3.202  **p=0.001** | Z=-3.609  **p≤0.001** | Z=-3.268  **p=0.001** |

Table E: ‘U shaped inverted’ (N = 279)

| **U shaped**  **inverted** | ***M.m.m.1*** | ***M.m.m.2*** | ***M.m.m.3*** | ***M.m.m.4*** | **Hybrid 1** | **Hybrid 2** | ***M.m.d.*** |
| --- | --- | --- | --- | --- | --- | --- | --- |
| ***M.m.m.2*** | Z=-1.977  p=0.048 |  |  |  |  |  |  |
| ***M.m.m.3*** | Z=-2.354  **p=0.019** | Z=-3.762  **p≤0.001** |  |  |  |  |  |
| ***M.m.m.4*** | Z=-0.077  p=0.939 | Z=-1.976  p=0.048 | Z=-2.353  **p=0.019** |  |  |  |  |
| **Hybrid 1** | Z=-1.427  p=0.154 | Z=-2.414  **p=0.016** | Z=-0.158  p=0.874 | Z=-1.422  p=0.155 |  |  |  |
| **Hybrid 2** |  |  |  |  |  |  |  |
| ***M.m.d.*** | Z=-2.832  **p=0.005** | Z=-1.140  p=0.254 | Z=-2.833  **p=0.005** | Z=-0.647  p=0.518 | Z=-1.799  p=0.072 |  |  |
| ***M.s.*** | Z=-3.526  **p≤0.001** | Z=-2.045  p=0.041 | Z=-3.869  **p≤0.001** | Z=-3.224  **p=0.001** | Z=-2.969  **p=0.003** |  | Z=-2.551  **p=0.011** |

Table F: ‘2-Frequency-Step’ (N = 67)

| **2-Frequency-step** | ***M.m.m.1*** | ***M.m.m.2*** | ***M.m.m.3*** | ***M.m.m.4*** | **Hybrid 1** | **Hybrid 2** | ***M.m.d.*** |
| --- | --- | --- | --- | --- | --- | --- | --- |
| ***M.m.m.2*** |  |  |  |  |  |  |  |
| ***M.m.m.3*** |  |  |  |  |  |  |  |
| ***M.m.m.4*** |  |  | Z=-0.398  p=0.691 |  |  |  |  |
| **Hybrid 1** |  |  | Z=-1.022  p=0.307 | Z=-0.694  p=0.488 |  |  |  |
| **Hybrid 2** |  |  | Z=-0.848  p=0.397 | Z=-0.514  p=0.607 | Z=-0.175  p=0.861 |  |  |
| ***M.m.d.*** |  |  | Z=-1.403  p=0.161 | Z=-1.178  p=0.239 | Z=-0.891  p=0.373 | Z=-0.797  p=0.426 |  |
| ***M.s.*** |  |  | Z=-1.989  p=0.047 | Z=-1.741  p=0.082 | Z=-1.071  p=0.284 | Z=-1.117  p=0.264 | Z=-0.360  p=0.719 |
